# 1-{2-Phenyl-2-[4-(trifluoro­meth­yl)­benzyl­oxy]eth­yl}-1*H*-benzimidazole

**DOI:** 10.1107/S1600536808024033

**Published:** 2008-07-31

**Authors:** Özden Özel Güven, Taner Erdoğan, Simon J. Coles, Tuncer Hökelek

**Affiliations:** a Zonguldak Karaelmas University, Department of Chemistry, 67100 Zonguldak, Turkey; b Southampton University, Department of Chemistry, Southampton SO17 1BJ, England; c Hacettepe University, Department of Physics, 06800 Beytepe, Ankara, Turkey

## Abstract

The asymmetric unit of the crystal structure of the title compound, C_23_H_19_F_3_N_2_O, contains two independent mol­ecules. In the two mol­ecules the planar benzimidazole ring systems are oriented with respect to the phen­yl/trifluoro­methyl­benzene rings at dihedral angles of 9.62 (6)/78.63 (7) and 2.53 (8)/83.83 (9)°. In the crystal structure, inter­molecular C—H⋯N hydrogen bonds link the mol­ecules into *R*_2_^2^(6) dimers. The mol­ecules are elongated along [001] and stacked along the *b* axis.

## Related literature

For general background, see: Brammer & Feczko (1988 ▶); Özel Güven *et al.* (2007*a* ▶,*b* ▶). For related literature, see: Song & Shin (1998 ▶); Freer *et al.* (1986 ▶); Peeters *et al.* (1996 ▶, 1979*a* ▶,*b* ▶); Caira *et al.* (2004 ▶); Özel Güven *et al.* (2008*a* ▶,*b* ▶). For ring motif details, see: Bernstein *et al.* (1995 ▶).
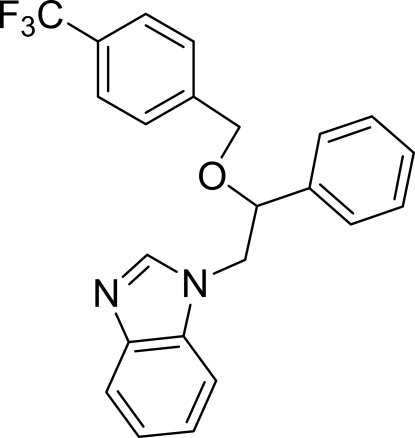


## Experimental

### 

#### Crystal data

C_23_H_19_F_3_N_2_O
*M* *_r_* = 396.40
Monoclinic, 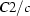

*a* = 39.3006 (15) Å
*b* = 9.5834 (2) Å
*c* = 23.0120 (9) Å
β = 113.668 (1)°
*V* = 7938.1 (5) Å^3^
*Z* = 16
Mo *K*α radiation
μ = 0.10 mm^−1^
*T* = 120 (2) K
0.35 × 0.2 × 0.14 mm

#### Data collection

Bruker Nonius KappaCCD diffractometer
Absorption correction: multi-scan (*SADABS*; Sheldrick, 2007 ▶) *T*_min_ = 0.974, *T*_max_ = 0.984
28014 measured reflections
8735 independent reflections
5066 reflections with *I* > 2σ(*I*)
*R*_int_ = 0.072

#### Refinement

*R*[*F* ^2^ > 2σ(*F* ^2^)] = 0.081
*wR*(*F* ^2^) = 0.227
*S* = 1.06
8735 reflections
524 parameters
H-atom parameters constrained
Δρ_max_ = 0.62 e Å^−3^
Δρ_min_ = −0.59 e Å^−3^

### 

Data collection: *COLLECT* (Nonius, 1998 ▶); cell refinement: *DENZO* (Otwinowski & Minor, 1997 ▶) and *COLLECT*; data reduction: *DENZO* and *COLLECT*; program(s) used to solve structure: *SHELXS97* (Sheldrick, 2008 ▶); program(s) used to refine structure: *SHELXL97* (Sheldrick, 2008 ▶); molecular graphics: *ORTEP-3 for Windows* (Farrugia, 1997 ▶); software used to prepare material for publication: *WinGX* (Farrugia, 1999 ▶).

## Supplementary Material

Crystal structure: contains datablocks I, global. DOI: 10.1107/S1600536808024033/xu2445sup1.cif

Structure factors: contains datablocks I. DOI: 10.1107/S1600536808024033/xu2445Isup2.hkl

Additional supplementary materials:  crystallographic information; 3D view; checkCIF report

## Figures and Tables

**Table 1:** Hydrogen-bond geometry (Å, °)

*D*—H⋯*A*	*D*—H	H⋯*A*	*D*⋯*A*	*D*—H⋯*A*
C1—H1⋯N2′^i^	0.93	2.45	3.210 (5)	139
C1′—H1′⋯N2^ii^	0.93	2.47	3.215 (5)	138

Symmetry codes: (i) 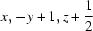; (ii) 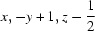.

**Fig. 1.**
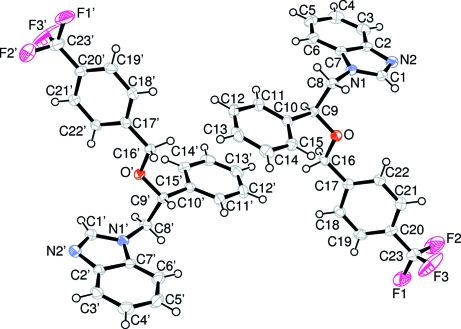
The molecular structure of the title molecule with the atom-numbering scheme. Displacement ellipsoids are drawn at the 50% probability level.

**Fig. 2.**
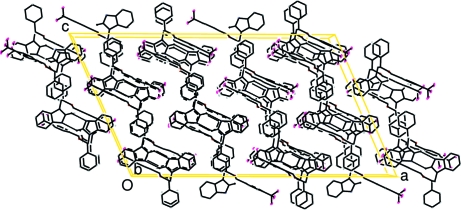
A packing diagram of the title compound. Hydrogen bonds are shown as dashed lines.

## supplementary crystallographic information

### Comment

In recent years, there has been increasing interest in synthesis of heterocyclic compounds having biological and commercial importances. Clotrimazole (Song & Shin, 1998), econazole (Freer *et al.*, 1986), ketoconazole (Peeters *et al.*, 1979*a*) and miconazole (Peeters *et al.*, 1979*b*) are well known imidazole ring containing, while itraconazole (Peeters *et al.*, 1996) and fluconazole (Caira *et al.*, 2004) are 1*H*-1,2,4-triazole ring containing, azole derivatives. They have been developed for clinical uses as antifungal agents (Brammer & Feczko, 1988). Lately, similar structures to miconazole and econazole have been reported to show antibacterial activity more than antifungal activity (Özel Güven *et al.*, 2007*a*,*b*). In these structures, benzimidazole ring has been found in place of the imidazole ring of miconazole and econazole. Recently, we reported the crystal structures of furyl (Özel Güven *et al.*, 2008*a*) and phenyl (Özel Güven *et al.*, 2008*b*) substituted compounds, and we report herein the crystal structure of title benzimidazole derivative.

The asymmetric unit of the crystal structure of the title compound (Fig. 1) contains two independent molecules, in which the bond lengths and angles are generally within normal ranges. The planar benzimidazole ring systems are oriented with respect to the phenyl and trifluoromethylbenzene rings at dihedral angles of 9.62 (6)°, 78.63 (7)° and 2.53 (8)°, 83.83 (9)° for unprimed and primed molecules, respectively. Atoms C8, C9, C16 and C8', C9', C16' are -0.102 (2), 0.093 (2), -0.008 (3) and 0.122 (3), -0.100 (3), -0.003 (3) Å away from the ring planes of the corresponding benzimidazole, phenyl and trifluoromethylbenzene, respectively. So, they are nearly coplanar with the attached rings. The trifluoromethylbenzene rings are oriented with respect to the phenyl rings at dihedral angles of 75.97 (9)° and 86.13 (9)° for unprimed and primed molecules, respectively.

In the crystal structure, intermolecular C—H···N hydrogen bonds (Table 1) link the molecules to form a *R*_2_^2^(6) ring motif (Bernstein *et al.*, 1995), in which they may be effective in the stabilization of the structure. The molecules are elongated along [001], and stacked along the *b* axis (Fig. 2).

### Experimental

The title compound was synthesized by the reaction of 2-(1*H*-benzimidazol-1-yl)-1-phenylethanol (Özel Güven *et al.*, 2007*a*) with NaH and appropriate benzyl halide. To the solution of alcohol (300 mg, 1.259 mmol) in DMF (2.4 ml) was added NaH (63 mg, 1.574 mmol) in small fractions. The appropriate benzyl halide (300 mg, 1.259 mmol) in DMF (1.2 ml) was added dropwise. The mixture was stirred at room temperature for 2 h and excess hydride was decomposed with a small amount of methyl alcohol. After evaporation to dryness under reduced pressure, the crude residue was suspended with water and extracted with methylene chloride. The organic layer was dried over anhydrous sodium sulfate, and then evaporated to dryness. The crude residue was purified by chromatography on a silica-gel column using chloroform–methanol as eluent. Crystals suitable for X-ray analysis were obtained by the recrystallization of the ether from a mixture of hexane/ethyl acetate (1:2) (yield; 296 mg, 59%).

### Refinement

H atoms were positioned geometrically, with C—H = 0.93, 0.98 and 0.97 Å for aromatic, methine and methylene H, respectively, and constrained to ride on their parent atoms with *U*_iso_(H) = 1.2*U*_eq_(C).

### Crystal data

**Table:**

C_23_H_19_F_3_N_2_O	*F*_000_ = 3296
*M**_r_* = 396.40	*D*_x_ = 1.327 Mg m^−^^3^
Monoclinic, *C*2/*c*	Mo *K*α radiation λ = 0.71073 Å
Hall symbol: -C 2yc	Cell parameters from 8156 reflections
*a* = 39.3006 (15) Å	θ = 2.9–27.5º
*b* = 9.5834 (2) Å	µ = 0.10 mm^−^^1^
*c* = 23.0120 (9) Å	*T* = 120 (2) K
β = 113.6680 (10)º	Shard, colourless
*V* = 7938.1 (5) Å^3^	0.35 × 0.2 × 0.14 mm
*Z* = 16	

### Data collection

**Table:**

Bruker Nonius KappaCCD diffractometer	8735 independent reflections
Radiation source: fine-focus sealed tube	5066 reflections with *I* > 2σ(*I*)
Monochromator: graphite	*R*_int_ = 0.072
Detector resolution: 9.091 pixels mm^-1^	θ_max_ = 27.5º
*T* = 120(2) K	θ_min_ = 3.1º
φ and ω scans	*h* = −50→39
Absorption correction: multi-scan(SADABS; Sheldrick, 2007)	*k* = −10→11
*T*_min_ = 0.974, *T*_max_ = 0.984	*l* = −29→29
28014 measured reflections	

### Refinement

**Table:**

Refinement on *F*^2^	Hydrogen site location: inferred from neighbouring sites
Least-squares matrix: full	H-atom parameters constrained
*R*[*F*^2^ > 2σ(*F*^2^)] = 0.081	*w* = 1/[σ^2^(*F*_o_^2^) + (0.117*P*)^2^ + 2.4537*P*] where *P* = (*F*_o_^2^ + 2*F*_c_^2^)/3
*wR*(*F*^2^) = 0.227	(Δ/σ)_max_ < 0.001
*S* = 1.06	Δρ_max_ = 0.62 e Å^−^^3^
8735 reflections	Δρ_min_ = −0.59 e Å^−^^3^
524 parameters	Extinction correction: SHELXL97 (Sheldrick, 2008), Fc^*^=kFc[1+0.001xFc^2^λ^3^/sin(2θ)]^-1/4^
Primary atom site location: structure-invariant direct methods	Extinction coefficient: 0.0062 (4)
Secondary atom site location: difference Fourier map	

### Special details

**Table:**

Geometry. All e.s.d.'s (except the e.s.d. in the dihedral angle between two l.s. planes) are estimated using the full covariance matrix. The cell e.s.d.'s are taken into account individually in the estimation of e.s.d.'s in distances, angles and torsion angles; correlations between e.s.d.'s in cell parameters are only used when they are defined by crystal symmetry. An approximate (isotropic) treatment of cell e.s.d.'s is used for estimating e.s.d.'s involving l.s. planes.
Refinement. Refinement of *F*^2^ against ALL reflections. The weighted *R*-factor *wR* and goodness of fit *S* are based on *F*^2^, conventional *R*-factors *R* are based on *F*, with *F* set to zero for negative *F*^2^. The threshold expression of *F*^2^ > σ(*F*^2^) is used only for calculating *R*-factors(gt) *etc*. and is not relevant to the choice of reflections for refinement. *R*-factors based on *F*^2^ are statistically about twice as large as those based on *F*, and *R*- factors based on ALL data will be even larger.

### Fractional atomic coordinates and isotropic or equivalent isotropic displacement parameters (Å^2^)

**Table:**

	*x*	*y*	*z*	*U*_iso_*/*U*_eq_	
F1	−0.00131 (8)	0.4243 (2)	0.64336 (15)	0.0898 (10)	
F2	0.01662 (7)	0.6106 (3)	0.69271 (12)	0.0802 (8)	
F3	−0.02789 (6)	0.6169 (3)	0.60373 (13)	0.0929 (10)	
O	0.10852 (5)	0.78467 (16)	0.49859 (8)	0.0227 (4)	
N1	0.15853 (6)	1.0225 (2)	0.52907 (10)	0.0225 (5)	
N2	0.17053 (6)	1.1149 (2)	0.62514 (11)	0.0299 (6)	
C1	0.14498 (8)	1.0792 (3)	0.56973 (13)	0.0268 (6)	
H1	0.1197	1.0917	0.5591	0.032*	
C2	0.20392 (7)	1.0775 (3)	0.62118 (13)	0.0251 (6)	
C3	0.24023 (8)	1.0885 (3)	0.66600 (14)	0.0329 (7)	
H3	0.2455	1.1252	0.7061	0.039*	
C4	0.26834 (8)	1.0430 (3)	0.64890 (15)	0.0386 (8)	
H4	0.2928	1.0494	0.6782	0.046*	
C5	0.26089 (8)	0.9874 (3)	0.58851 (15)	0.0347 (7)	
H5	0.2805	0.9588	0.5786	0.042*	
C6	0.22489 (7)	0.9744 (3)	0.54353 (14)	0.0278 (6)	
H6	0.2197	0.9371	0.5036	0.033*	
C7	0.19684 (7)	1.0200 (2)	0.56120 (13)	0.0235 (6)	
C8	0.13789 (7)	0.9648 (3)	0.46651 (12)	0.0240 (6)	
H8A	0.1507	0.9866	0.4393	0.029*	
H8B	0.1135	1.0077	0.4483	0.029*	
C9	0.13351 (7)	0.8071 (2)	0.46865 (12)	0.0218 (6)	
H9	0.1576	0.7659	0.4949	0.026*	
C10	0.12009 (7)	0.7464 (2)	0.40188 (12)	0.0229 (6)	
C11	0.14588 (8)	0.7186 (3)	0.37625 (14)	0.0295 (6)	
H11	0.1711	0.7313	0.4008	0.035*	
C12	0.13429 (9)	0.6719 (3)	0.31405 (15)	0.0339 (7)	
H12	0.1517	0.6544	0.2969	0.041*	
C13	0.09678 (9)	0.6513 (3)	0.27756 (14)	0.0346 (7)	
H13	0.0889	0.6201	0.2359	0.042*	
C14	0.07127 (8)	0.6773 (3)	0.30323 (14)	0.0336 (7)	
H14	0.0461	0.6628	0.2788	0.040*	
C15	0.08258 (8)	0.7250 (3)	0.36512 (13)	0.0274 (6)	
H15	0.0651	0.7426	0.3819	0.033*	
C16	0.10907 (8)	0.6442 (2)	0.51903 (13)	0.0253 (6)	
H16A	0.1338	0.6210	0.5498	0.030*	
H16B	0.1031	0.5817	0.4831	0.030*	
C17	0.08146 (7)	0.6250 (2)	0.54841 (12)	0.0232 (6)	
C18	0.07287 (8)	0.4897 (3)	0.56064 (13)	0.0279 (6)	
H18	0.0841	0.4140	0.5502	0.033*	
C19	0.04785 (8)	0.4669 (3)	0.58808 (14)	0.0306 (7)	
H19	0.0423	0.3763	0.5959	0.037*	
C20	0.03112 (7)	0.5793 (3)	0.60393 (14)	0.0275 (6)	
C21	0.03939 (8)	0.7142 (3)	0.59224 (13)	0.0280 (6)	
H21	0.0280	0.7895	0.6026	0.034*	
C22	0.06473 (7)	0.7369 (3)	0.56503 (12)	0.0242 (6)	
H22	0.0705	0.8276	0.5579	0.029*	
C23	0.00440 (9)	0.5567 (3)	0.63439 (16)	0.0389 (7)	
F1'	0.24560 (6)	0.5820 (2)	0.09554 (12)	0.0674 (7)	
F2'	0.23310 (10)	0.3851 (3)	0.05370 (16)	0.1134 (13)	
F3'	0.27574 (8)	0.4034 (4)	0.14267 (18)	0.1280 (15)	
O'	0.13628 (5)	0.20393 (16)	0.23713 (9)	0.0240 (4)	
N1'	0.08787 (6)	−0.0357 (2)	0.20786 (10)	0.0248 (5)	
N2'	0.07698 (7)	−0.1235 (2)	0.11146 (11)	0.0294 (5)	
C1'	0.10222 (8)	−0.0896 (3)	0.16771 (13)	0.0277 (6)	
H1'	0.1276	−0.1013	0.1790	0.033*	
C2'	0.04340 (8)	−0.0877 (3)	0.11429 (13)	0.0262 (6)	
C3'	0.00741 (8)	−0.0977 (3)	0.06867 (14)	0.0320 (7)	
H3'	0.0027	−0.1349	0.0289	0.038*	
C4'	−0.02128 (8)	−0.0513 (3)	0.08355 (15)	0.0371 (7)	
H4'	−0.0456	−0.0566	0.0532	0.045*	
C5'	−0.01454 (9)	0.0040 (3)	0.14381 (16)	0.0398 (8)	
H5'	−0.0345	0.0345	0.1525	0.048*	
C6'	0.02114 (8)	0.0140 (3)	0.19057 (15)	0.0341 (7)	
H6'	0.0257	0.0499	0.2305	0.041*	
C7'	0.04969 (7)	−0.0325 (3)	0.17457 (13)	0.0254 (6)	
C8'	0.10849 (8)	0.0217 (3)	0.27057 (13)	0.0262 (6)	
H8C	0.1330	−0.0202	0.2885	0.031*	
H8D	0.0959	−0.0018	0.2978	0.031*	
C9'	0.11245 (7)	0.1798 (3)	0.26909 (13)	0.0235 (6)	
H9'	0.0881	0.2213	0.2446	0.028*	
C10'	0.12750 (7)	0.2370 (2)	0.33608 (12)	0.0228 (6)	
C11'	0.10322 (8)	0.2853 (3)	0.36199 (14)	0.0287 (6)	
H11'	0.0779	0.2887	0.3370	0.034*	
C12'	0.11659 (9)	0.3284 (3)	0.42489 (15)	0.0356 (7)	
H12'	0.1002	0.3594	0.4420	0.043*	
C13'	0.15418 (9)	0.3251 (3)	0.46192 (14)	0.0357 (7)	
H13'	0.1631	0.3544	0.5039	0.043*	
C14'	0.17852 (8)	0.2787 (3)	0.43688 (13)	0.0329 (7)	
H14'	0.2039	0.2774	0.4621	0.040*	
C15'	0.16558 (7)	0.2334 (3)	0.37403 (13)	0.0275 (6)	
H15'	0.1822	0.2010	0.3575	0.033*	
C16'	0.13615 (7)	0.3468 (2)	0.21945 (13)	0.0225 (6)	
H16C	0.1420	0.4057	0.2565	0.027*	
H16D	0.1116	0.3719	0.1886	0.027*	
C17'	0.16427 (7)	0.3700 (2)	0.19148 (12)	0.0221 (6)	
C18'	0.17043 (7)	0.5057 (3)	0.17587 (13)	0.0250 (6)	
H18'	0.1571	0.5791	0.1831	0.030*	
C19'	0.19571 (8)	0.5330 (3)	0.15004 (14)	0.0292 (6)	
H19'	0.1993	0.6240	0.1397	0.035*	
C20'	0.21595 (8)	0.4242 (3)	0.13926 (14)	0.0296 (6)	
C21'	0.21006 (8)	0.2884 (3)	0.15450 (14)	0.0284 (6)	
H21'	0.2235	0.2153	0.1474	0.034*	
C22'	0.18436 (7)	0.2614 (3)	0.18011 (12)	0.0242 (6)	
H22'	0.1805	0.1701	0.1898	0.029*	
C23'	0.24321 (10)	0.4507 (3)	0.11059 (19)	0.0450 (9)	

### Atomic displacement parameters (Å^2^)

**Table:**

	*U*^11^	*U*^22^	*U*^33^	*U*^12^	*U*^13^	*U*^23^
F1	0.112 (2)	0.0504 (13)	0.161 (3)	−0.0173 (12)	0.111 (2)	0.0023 (14)
F2	0.0848 (18)	0.120 (2)	0.0586 (16)	−0.0341 (15)	0.0520 (15)	−0.0158 (14)
F3	0.0403 (13)	0.159 (3)	0.092 (2)	0.0345 (14)	0.0405 (14)	0.0613 (18)
O	0.0255 (10)	0.0218 (9)	0.0217 (10)	−0.0001 (7)	0.0105 (8)	0.0015 (7)
N1	0.0235 (12)	0.0243 (11)	0.0194 (12)	−0.0039 (8)	0.0085 (10)	−0.0010 (8)
N2	0.0315 (14)	0.0300 (12)	0.0298 (14)	−0.0060 (9)	0.0141 (12)	−0.0046 (10)
C1	0.0301 (15)	0.0251 (13)	0.0290 (16)	−0.0034 (10)	0.0159 (13)	−0.0008 (11)
C2	0.0263 (14)	0.0235 (12)	0.0236 (15)	−0.0056 (10)	0.0081 (12)	0.0014 (10)
C3	0.0360 (17)	0.0343 (15)	0.0219 (16)	−0.0090 (12)	0.0050 (13)	−0.0018 (11)
C4	0.0251 (16)	0.0440 (17)	0.0346 (19)	−0.0054 (12)	−0.0007 (14)	0.0040 (14)
C5	0.0222 (15)	0.0409 (16)	0.0425 (19)	−0.0034 (12)	0.0143 (14)	0.0031 (13)
C6	0.0256 (15)	0.0330 (14)	0.0253 (16)	−0.0038 (11)	0.0105 (13)	−0.0004 (11)
C7	0.0232 (14)	0.0228 (12)	0.0228 (15)	−0.0042 (10)	0.0077 (12)	0.0013 (10)
C8	0.0232 (14)	0.0281 (13)	0.0191 (14)	−0.0044 (10)	0.0068 (12)	−0.0005 (10)
C9	0.0207 (13)	0.0262 (13)	0.0195 (14)	0.0005 (10)	0.0092 (11)	0.0022 (10)
C10	0.0276 (15)	0.0196 (12)	0.0226 (14)	0.0007 (10)	0.0112 (12)	0.0009 (10)
C11	0.0307 (16)	0.0262 (14)	0.0339 (17)	0.0028 (11)	0.0155 (13)	−0.0027 (11)
C12	0.0438 (19)	0.0306 (14)	0.0354 (18)	0.0029 (12)	0.0243 (16)	−0.0049 (12)
C13	0.053 (2)	0.0256 (14)	0.0233 (16)	0.0008 (12)	0.0134 (15)	−0.0025 (11)
C14	0.0322 (16)	0.0341 (15)	0.0261 (16)	−0.0039 (12)	0.0030 (13)	−0.0043 (12)
C15	0.0288 (15)	0.0285 (14)	0.0260 (16)	0.0007 (11)	0.0123 (13)	0.0004 (11)
C16	0.0338 (16)	0.0188 (12)	0.0247 (15)	0.0033 (10)	0.0133 (13)	0.0029 (10)
C17	0.0240 (14)	0.0262 (13)	0.0171 (14)	−0.0011 (10)	0.0059 (11)	0.0007 (10)
C18	0.0361 (16)	0.0213 (13)	0.0260 (16)	−0.0001 (10)	0.0122 (13)	0.0012 (10)
C19	0.0370 (17)	0.0234 (14)	0.0321 (17)	−0.0046 (11)	0.0148 (14)	0.0032 (11)
C20	0.0233 (14)	0.0316 (14)	0.0277 (16)	−0.0030 (11)	0.0103 (12)	0.0038 (11)
C21	0.0296 (15)	0.0290 (14)	0.0247 (15)	0.0056 (11)	0.0103 (13)	0.0035 (11)
C22	0.0260 (14)	0.0215 (12)	0.0229 (14)	0.0002 (10)	0.0075 (12)	0.0039 (10)
C23	0.0347 (18)	0.0411 (17)	0.043 (2)	−0.0033 (13)	0.0174 (16)	0.0042 (14)
F1'	0.0789 (16)	0.0480 (12)	0.106 (2)	−0.0099 (10)	0.0695 (16)	0.0101 (11)
F2'	0.175 (3)	0.106 (2)	0.126 (3)	−0.068 (2)	0.129 (3)	−0.0539 (19)
F3'	0.0609 (17)	0.180 (3)	0.179 (4)	0.0508 (19)	0.085 (2)	0.112 (3)
O'	0.0301 (10)	0.0218 (9)	0.0262 (11)	−0.0025 (7)	0.0176 (9)	0.0004 (7)
N1'	0.0301 (13)	0.0244 (11)	0.0215 (12)	−0.0065 (9)	0.0120 (11)	−0.0004 (9)
N2'	0.0337 (14)	0.0275 (12)	0.0287 (14)	−0.0057 (9)	0.0143 (11)	−0.0046 (9)
C1'	0.0311 (15)	0.0252 (13)	0.0289 (17)	−0.0043 (11)	0.0141 (13)	−0.0021 (11)
C2'	0.0336 (16)	0.0232 (13)	0.0255 (15)	−0.0082 (10)	0.0157 (13)	−0.0027 (10)
C3'	0.0385 (17)	0.0327 (15)	0.0260 (16)	−0.0113 (12)	0.0142 (14)	−0.0062 (12)
C4'	0.0242 (16)	0.0508 (18)	0.0309 (17)	−0.0106 (12)	0.0053 (13)	−0.0043 (14)
C5'	0.0297 (17)	0.0586 (19)	0.0368 (19)	−0.0087 (14)	0.0192 (15)	−0.0102 (15)
C6'	0.0327 (17)	0.0461 (17)	0.0269 (17)	−0.0099 (13)	0.0155 (14)	−0.0074 (13)
C7'	0.0281 (15)	0.0252 (13)	0.0249 (15)	−0.0098 (10)	0.0128 (12)	−0.0016 (11)
C8'	0.0284 (15)	0.0284 (14)	0.0203 (15)	−0.0072 (10)	0.0082 (12)	0.0001 (11)
C9'	0.0230 (14)	0.0283 (13)	0.0218 (14)	−0.0015 (10)	0.0115 (12)	0.0008 (10)
C10'	0.0267 (14)	0.0199 (12)	0.0224 (14)	−0.0020 (10)	0.0106 (12)	0.0002 (10)
C11'	0.0281 (15)	0.0270 (14)	0.0299 (16)	0.0002 (10)	0.0104 (13)	−0.0022 (11)
C12'	0.048 (2)	0.0298 (14)	0.0359 (18)	−0.0022 (12)	0.0246 (16)	−0.0089 (12)
C13'	0.051 (2)	0.0289 (14)	0.0263 (17)	−0.0093 (13)	0.0145 (15)	−0.0029 (12)
C14'	0.0312 (16)	0.0358 (15)	0.0237 (16)	−0.0106 (12)	0.0025 (13)	0.0032 (12)
C15'	0.0247 (15)	0.0308 (14)	0.0278 (16)	−0.0036 (11)	0.0112 (12)	0.0024 (11)
C16'	0.0242 (14)	0.0210 (12)	0.0231 (14)	−0.0011 (10)	0.0104 (12)	0.0004 (10)
C17'	0.0222 (14)	0.0258 (13)	0.0148 (13)	−0.0036 (10)	0.0036 (11)	−0.0022 (10)
C18'	0.0289 (15)	0.0218 (13)	0.0225 (15)	−0.0002 (10)	0.0085 (12)	−0.0034 (10)
C19'	0.0341 (16)	0.0252 (13)	0.0284 (16)	−0.0070 (11)	0.0127 (13)	0.0019 (11)
C20'	0.0324 (16)	0.0303 (14)	0.0302 (17)	−0.0083 (11)	0.0168 (13)	−0.0022 (11)
C21'	0.0302 (15)	0.0260 (14)	0.0328 (17)	−0.0030 (11)	0.0165 (13)	−0.0043 (11)
C22'	0.0275 (14)	0.0212 (12)	0.0241 (14)	−0.0031 (10)	0.0106 (12)	−0.0014 (10)
C23'	0.047 (2)	0.0380 (18)	0.063 (2)	−0.0060 (14)	0.0353 (19)	0.0021 (15)

### Geometric parameters (Å, °)

**Table:**

F1—C23	1.319 (3)	F1'—C23'	1.318 (3)
F2—C23	1.334 (4)	F2'—C23'	1.360 (4)
F3—C23	1.312 (4)	F3'—C23'	1.276 (4)
O—C9	1.424 (3)	O'—C9'	1.423 (3)
O—C16	1.424 (3)	O'—C16'	1.427 (3)
N1—C1	1.362 (3)	N1'—C1'	1.363 (3)
N1—C7	1.386 (3)	N1'—C7'	1.384 (3)
N1—C8	1.449 (3)	N1'—C8'	1.450 (3)
N2—C1	1.312 (4)	N2'—C1'	1.317 (4)
N2—C2	1.398 (4)	N2'—C2'	1.390 (4)
C1—H1	0.9300	C1'—H1'	0.9300
C2—C3	1.389 (4)	C2'—C3'	1.386 (4)
C3—C4	1.384 (4)	C2'—C7'	1.411 (4)
C3—H3	0.9300	C3'—C4'	1.377 (4)
C4—C5	1.405 (4)	C3'—H3'	0.9300
C4—H4	0.9300	C4'—C5'	1.407 (4)
C5—H5	0.9300	C4'—H4'	0.9300
C6—C5	1.383 (4)	C5'—H5'	0.9300
C6—H6	0.9300	C6'—C5'	1.386 (4)
C7—C2	1.407 (4)	C6'—H6'	0.9300
C7—C6	1.390 (4)	C7'—C6'	1.387 (4)
C8—H8A	0.9700	C8'—H8C	0.9700
C8—H8B	0.9700	C8'—H8D	0.9700
C9—C8	1.524 (3)	C9'—C8'	1.525 (3)
C9—C10	1.525 (4)	C9'—C10'	1.515 (4)
C9—H9	0.9800	C9'—H9'	0.9800
C10—C11	1.388 (4)	C10'—C11'	1.392 (4)
C10—C15	1.389 (4)	C10'—C15'	1.398 (4)
C11—C12	1.390 (4)	C11'—C12'	1.390 (4)
C11—H11	0.9300	C11'—H11'	0.9300
C12—C13	1.387 (4)	C12'—C13'	1.378 (4)
C12—H12	0.9300	C12'—H12'	0.9300
C13—H13	0.9300	C13'—H13'	0.9300
C14—C13	1.375 (4)	C14'—C13'	1.374 (4)
C14—H14	0.9300	C14'—H14'	0.9300
C15—C14	1.388 (4)	C15'—C14'	1.396 (4)
C15—H15	0.9300	C15'—H15'	0.9300
C16—H16A	0.9700	C16'—H16C	0.9700
C16—H16B	0.9700	C16'—H16D	0.9700
C17—C16	1.502 (4)	C17'—C16'	1.502 (4)
C17—C18	1.397 (3)	C17'—C22'	1.394 (4)
C17—C22	1.390 (4)	C18'—C17'	1.396 (3)
C18—C19	1.383 (4)	C18'—C19'	1.372 (4)
C18—H18	0.9300	C18'—H18'	0.9300
C19—H19	0.9300	C19'—C20'	1.393 (4)
C20—C19	1.385 (4)	C19'—H19'	0.9300
C20—C21	1.385 (4)	C20'—C23'	1.488 (4)
C20—C23	1.494 (4)	C21'—C20'	1.391 (4)
C21—H21	0.9300	C21'—H21'	0.9300
C22—C21	1.390 (4)	C22'—C21'	1.383 (4)
C22—H22	0.9300	C22'—H22'	0.9300
			
C16—O—C9	112.37 (18)	C9'—O'—C16'	112.04 (18)
C1—N1—C7	106.3 (2)	C1'—N1'—C7'	106.5 (2)
C1—N1—C8	128.2 (2)	C1'—N1'—C8'	126.9 (2)
C7—N1—C8	125.4 (2)	C7'—N1'—C8'	126.2 (2)
C1—N2—C2	104.0 (2)	C1'—N2'—C2'	104.3 (2)
N2—C1—N1	114.4 (2)	N2'—C1'—N1'	114.0 (2)
N2—C1—H1	122.8	N2'—C1'—H1'	123.0
N1—C1—H1	122.8	N1'—C1'—H1'	123.0
C3—C2—N2	130.1 (3)	C3'—C2'—N2'	130.3 (3)
C3—C2—C7	119.9 (3)	C3'—C2'—C7'	119.6 (3)
N2—C2—C7	110.1 (2)	N2'—C2'—C7'	110.1 (2)
C4—C3—C2	117.7 (3)	C4'—C3'—C2'	118.6 (3)
C4—C3—H3	121.2	C4'—C3'—H3'	120.7
C2—C3—H3	121.2	C2'—C3'—H3'	120.7
C3—C4—C5	121.9 (3)	C3'—C4'—C5'	121.2 (3)
C3—C4—H4	119.1	C3'—C4'—H4'	119.4
C5—C4—H4	119.1	C5'—C4'—H4'	119.4
C6—C5—C4	121.2 (3)	C6'—C5'—C4'	121.5 (3)
C6—C5—H5	119.4	C6'—C5'—H5'	119.2
C4—C5—H5	119.4	C4'—C5'—H5'	119.2
C5—C6—C7	116.5 (3)	C5'—C6'—C7'	116.5 (3)
C5—C6—H6	121.8	C5'—C6'—H6'	121.8
C7—C6—H6	121.8	C7'—C6'—H6'	121.8
N1—C7—C6	132.0 (2)	N1'—C7'—C6'	132.3 (3)
N1—C7—C2	105.2 (2)	N1'—C7'—C2'	105.0 (2)
C6—C7—C2	122.9 (2)	C6'—C7'—C2'	122.7 (3)
N1—C8—C9	111.8 (2)	N1'—C8'—C9'	112.1 (2)
N1—C8—H8A	109.2	N1'—C8'—H8C	109.2
C9—C8—H8A	109.2	C9'—C8'—H8C	109.2
N1—C8—H8B	109.2	N1'—C8'—H8D	109.2
C9—C8—H8B	109.2	C9'—C8'—H8D	109.2
H8A—C8—H8B	107.9	H8C—C8'—H8D	107.9
O—C9—C8	105.90 (19)	O'—C9'—C10'	113.5 (2)
O—C9—C10	113.8 (2)	O'—C9'—C8'	105.5 (2)
C8—C9—C10	110.1 (2)	C10'—C9'—C8'	109.8 (2)
O—C9—H9	109.0	O'—C9'—H9'	109.3
C8—C9—H9	109.0	C10'—C9'—H9'	109.3
C10—C9—H9	109.0	C8'—C9'—H9'	109.3
C11—C10—C15	119.4 (3)	C11'—C10'—C15'	119.1 (2)
C11—C10—C9	119.0 (2)	C11'—C10'—C9'	120.1 (2)
C15—C10—C9	121.5 (2)	C15'—C10'—C9'	120.6 (2)
C10—C11—C12	120.4 (3)	C12'—C11'—C10'	120.5 (3)
C10—C11—H11	119.8	C12'—C11'—H11'	119.8
C12—C11—H11	119.8	C10'—C11'—H11'	119.8
C13—C12—C11	119.9 (3)	C13'—C12'—C11'	120.0 (3)
C13—C12—H12	120.0	C13'—C12'—H12'	120.0
C11—C12—H12	120.0	C11'—C12'—H12'	120.0
C14—C13—C12	119.7 (3)	C14'—C13'—C12'	120.1 (3)
C14—C13—H13	120.2	C14'—C13'—H13'	119.9
C12—C13—H13	120.2	C12'—C13'—H13'	119.9
C13—C14—C15	120.8 (3)	C13'—C14'—C15'	120.7 (3)
C13—C14—H14	119.6	C13'—C14'—H14'	119.7
C15—C14—H14	119.6	C15'—C14'—H14'	119.7
C14—C15—C10	119.9 (3)	C14'—C15'—C10'	119.5 (3)
C14—C15—H15	120.1	C14'—C15'—H15'	120.2
C10—C15—H15	120.1	C10'—C15'—H15'	120.2
O—C16—C17	110.26 (19)	O'—C16'—C17'	110.20 (19)
O—C16—H16A	109.6	O'—C16'—H16C	109.6
C17—C16—H16A	109.6	C17'—C16'—H16C	109.6
O—C16—H16B	109.6	O'—C16'—H16D	109.6
C17—C16—H16B	109.6	C17'—C16'—H16D	109.6
H16A—C16—H16B	108.1	H16C—C16'—H16D	108.1
C22—C17—C18	118.8 (2)	C22'—C17'—C18'	118.5 (2)
C22—C17—C16	122.5 (2)	C22'—C17'—C16'	122.6 (2)
C18—C17—C16	118.7 (2)	C18'—C17'—C16'	118.8 (2)
C19—C18—C17	120.8 (2)	C19'—C18'—C17'	121.3 (2)
C19—C18—H18	119.6	C19'—C18'—H18'	119.4
C17—C18—H18	119.6	C17'—C18'—H18'	119.4
C18—C19—C20	119.8 (2)	C18'—C19'—C20'	120.0 (2)
C18—C19—H19	120.1	C18'—C19'—H19'	120.0
C20—C19—H19	120.1	C20'—C19'—H19'	120.0
C19—C20—C21	120.1 (2)	C21'—C20'—C19'	119.4 (2)
C19—C20—C23	120.5 (2)	C21'—C20'—C23'	119.6 (3)
C21—C20—C23	119.3 (2)	C19'—C20'—C23'	121.0 (2)
C20—C21—C22	120.0 (2)	C22'—C21'—C20'	120.4 (2)
C20—C21—H21	120.0	C22'—C21'—H21'	119.8
C22—C21—H21	120.0	C20'—C21'—H21'	119.8
C17—C22—C21	120.5 (2)	C21'—C22'—C17'	120.4 (2)
C17—C22—H22	119.8	C21'—C22'—H22'	119.8
C21—C22—H22	119.8	C17'—C22'—H22'	119.8
F3—C23—F1	108.6 (3)	F3'—C23'—F1'	109.3 (3)
F3—C23—F2	104.5 (3)	F3'—C23'—F2'	103.3 (3)
F1—C23—F2	103.3 (3)	F1'—C23'—F2'	102.2 (3)
F3—C23—C20	113.2 (3)	F3'—C23'—C20'	114.5 (3)
F1—C23—C20	114.1 (3)	F1'—C23'—C20'	114.8 (3)
F2—C23—C20	112.3 (3)	F2'—C23'—C20'	111.3 (3)
			
C16—O—C9—C8	−164.6 (2)	C16'—O'—C9'—C10'	−72.8 (3)
C16—O—C9—C10	74.4 (3)	C16'—O'—C9'—C8'	167.0 (2)
C9—O—C16—C17	−178.3 (2)	C9'—O'—C16'—C17'	174.7 (2)
C7—N1—C1—N2	0.3 (3)	C7'—N1'—C1'—N2'	−0.7 (3)
C8—N1—C1—N2	175.7 (2)	C8'—N1'—C1'—N2'	−174.4 (2)
C1—N1—C7—C6	−179.8 (3)	C1'—N1'—C7'—C6'	−178.4 (3)
C8—N1—C7—C6	4.7 (4)	C8'—N1'—C7'—C6'	−4.7 (4)
C1—N1—C7—C2	0.0 (3)	C1'—N1'—C7'—C2'	0.3 (3)
C8—N1—C7—C2	−175.6 (2)	C8'—N1'—C7'—C2'	174.1 (2)
C1—N1—C8—C9	−95.0 (3)	C1'—N1'—C8'—C9'	94.8 (3)
C7—N1—C8—C9	79.6 (3)	C7'—N1'—C8'—C9'	−77.7 (3)
C2—N2—C1—N1	−0.5 (3)	C2'—N2'—C1'—N1'	0.8 (3)
C1—N2—C2—C3	−178.9 (3)	C1'—N2'—C2'—C3'	178.9 (3)
C1—N2—C2—C7	0.5 (3)	C1'—N2'—C2'—C7'	−0.6 (3)
N2—C2—C3—C4	−179.8 (3)	N2'—C2'—C3'—C4'	−178.6 (3)
C7—C2—C3—C4	0.8 (4)	C7'—C2'—C3'—C4'	0.8 (4)
C2—C3—C4—C5	0.0 (4)	C3'—C2'—C7'—N1'	−179.4 (2)
C3—C4—C5—C6	−0.7 (4)	N2'—C2'—C7'—N1'	0.1 (3)
C7—C6—C5—C4	0.5 (4)	C3'—C2'—C7'—C6'	−0.5 (4)
N1—C7—C2—C3	179.1 (2)	N2'—C2'—C7'—C6'	179.0 (2)
C6—C7—C2—C3	−1.0 (4)	C2'—C3'—C4'—C5'	−0.6 (4)
N1—C7—C2—N2	−0.3 (3)	C3'—C4'—C5'—C6'	0.0 (5)
C6—C7—C2—N2	179.5 (2)	C7'—C6'—C5'—C4'	0.4 (4)
N1—C7—C6—C5	−179.9 (2)	N1'—C7'—C6'—C5'	178.4 (3)
C2—C7—C6—C5	0.4 (4)	C2'—C7'—C6'—C5'	−0.1 (4)
O—C9—C8—N1	70.0 (3)	O'—C9'—C8'—N1'	−68.2 (3)
C10—C9—C8—N1	−166.6 (2)	C10'—C9'—C8'—N1'	169.3 (2)
O—C9—C10—C11	−158.4 (2)	O'—C9'—C10'—C11'	148.2 (2)
O—C9—C10—C15	24.8 (3)	C8'—C9'—C10'—C11'	−94.0 (3)
C8—C9—C10—C11	82.9 (3)	O'—C9'—C10'—C15'	−36.1 (3)
C8—C9—C10—C15	−93.8 (3)	C8'—C9'—C10'—C15'	81.6 (3)
C9—C10—C11—C12	−175.8 (2)	C15'—C10'—C11'—C12'	−0.5 (4)
C15—C10—C11—C12	1.0 (4)	C9'—C10'—C11'—C12'	175.3 (2)
C9—C10—C15—C14	176.2 (2)	C11'—C10'—C15'—C14'	−0.3 (4)
C11—C10—C15—C14	−0.5 (4)	C9'—C10'—C15'—C14'	−176.0 (2)
C10—C11—C12—C13	−0.8 (4)	C10'—C11'—C12'—C13'	0.8 (4)
C11—C12—C13—C14	0.0 (4)	C11'—C12'—C13'—C14'	−0.3 (4)
C15—C14—C13—C12	0.5 (4)	C15'—C14'—C13'—C12'	−0.5 (4)
C10—C15—C14—C13	−0.3 (4)	C10'—C15'—C14'—C13'	0.8 (4)
C22—C17—C16—O	−13.1 (4)	C22'—C17'—C16'—O'	5.4 (3)
C18—C17—C16—O	168.3 (2)	C18'—C17'—C16'—O'	−175.0 (2)
C22—C17—C18—C19	0.8 (4)	C18'—C17'—C22'—C21'	0.6 (4)
C16—C17—C18—C19	179.5 (2)	C16'—C17'—C22'—C21'	−179.7 (2)
C18—C17—C22—C21	−1.2 (4)	C19'—C18'—C17'—C22'	−0.2 (4)
C16—C17—C22—C21	−179.9 (2)	C19'—C18'—C17'—C16'	−179.8 (2)
C17—C18—C19—C20	−0.2 (4)	C17'—C18'—C19'—C20'	−0.4 (4)
C21—C20—C19—C18	0.1 (4)	C18'—C19'—C20'—C21'	0.4 (4)
C23—C20—C19—C18	−179.2 (3)	C18'—C19'—C20'—C23'	179.3 (3)
C19—C20—C21—C22	−0.5 (4)	C21'—C20'—C23'—F3'	−55.0 (5)
C23—C20—C21—C22	178.8 (3)	C19'—C20'—C23'—F3'	126.2 (4)
C19—C20—C23—F1	−0.6 (4)	C21'—C20'—C23'—F1'	177.3 (3)
C21—C20—C23—F1	−179.9 (3)	C19'—C20'—C23'—F1'	−1.5 (5)
C19—C20—C23—F2	116.5 (3)	C21'—C20'—C23'—F2'	61.8 (4)
C21—C20—C23—F2	−62.8 (4)	C19'—C20'—C23'—F2'	−117.0 (3)
C19—C20—C23—F3	−125.4 (3)	C22'—C21'—C20'—C19'	0.0 (4)
C21—C20—C23—F3	55.3 (4)	C22'—C21'—C20'—C23'	−178.8 (3)
C17—C22—C21—C20	1.1 (4)	C17'—C22'—C21'—C20'	−0.6 (4)

### Hydrogen-bond geometry (Å, °)

**Table:**

*D*—H···*A*	*D*—H	H···*A*	*D*···*A*	*D*—H···*A*
C1—H1···N2'^i^	0.93	2.45	3.210 (5)	139
C1'—H1'···N2^ii^	0.93	2.47	3.215 (5)	138

Symmetry codes: (i) *x*, −*y*+1, *z*+1/2; (ii) *x*, −*y*+1, *z*−1/2.
